# Recovery of Cerium Salts from Sewage Sludge Resulting from the Coagulation of Brewery Wastewater with Recycled Cerium Coagulant

**DOI:** 10.3390/ma17040938

**Published:** 2024-02-17

**Authors:** Paweł Lejwoda, Barbara Białecka, Krzysztof Barbusiński, Maciej Thomas

**Affiliations:** 1 Department of Energy Saving and Air Protection, Central Mining Institute in Katowice, Plac Gwarków 1, 40-166 Katowice, Poland; plejwoda@gig.eu; 2 Department of Environmental Monitoring, Central Mining Institute in Katowice, Plac Gwarków 1, 40-166 Katowice, Poland; bbialecka@gig.eu; 3 Department of Water and Wastewater Engineering, Silesian University of Technology, Konarskiego 18, 44-100 Gliwice, Poland; krzysztof.barbusinski@polsl.pl; 4 Faculty of Environmental Engineering and Energy, Cracow University of Technology, Warszawska 24, 31-155 Cracow, Poland

**Keywords:** brewery wastewater, response surface methodology, central composite design, sewage sludge, cerium coagulants

## Abstract

Due to the high cost and limited sources of cerium coagulants, it is extremely important to take measures to recycle this raw material. This paper presents the new possibility of recovering cerium(III) chloride, cerium(III) sulphate, cerium(IV) sulphate, and potentially phosphate from sewage sludge (101.5 g/kg Ce and 22.2 g/kg total P) through a brewery wastewater treatment process using recycled CeCl_3_ as a coagulant. In order to recover the Ce and P, the sludge was subjected to extraction using an HCl solution. Optimal process conditions were determined by means of central composite design and response surface methodology (CCD/RSM) for three input parameters (HCl mass, reaction time, and extractant volume). Under optimal conditions (0.35 g HCl per 1 g of sludge, 40 min reaction time, extractant volume of 25 mL per 1 g of sludge), the highest efficiency obtained was 99.6% and 97.5% for Ce and P, respectively. Cerium(III) oxalate as Ce_2_(C_2_O_4_)_3_∙10H_2_O was precipitated from the obtained solution using H_2_C_2_O_4_ (99.97%) and decomposed into CeO_2_ (at 350 °C), which was afterwards subjected to a reaction with HCl (30%, m/m) and H_2_O_2_ (30%, m/m), which led to the crystallisation of CeCl_3_∙7H_2_O with a purity of 98.6% and a yield of 97.0%. The obtained CeO_2_ was also subjected to a reaction with H_2_SO_4_ (96%, m/m) and H_2_O_2_ (30%, m/m), which produced Ce_2_(SO_4_)_3_ with a yield of 97.4%. The CeO_2_ was also subjected to a reaction with only H_2_SO_4_ (96%, m/m), which produced Ce(SO_4_)_2_ with a yield of 98.3%. The filtrate obtained after filtering the Ce_2_(C_2_O_4_)_3_∙10H_2_O contained 570 mg/L of P, which enabled its use as a source of phosphorus compounds. The presented processes of Ce and potentially P recovery from sewage sludge originating from brewery wastewater contribute to the idea of a circular economy.

## 1. Introduction

The decreasing supply of potable water in the world is a consequence of numerous negative factors, including the increasing population, developing industry, and uncontrolled pollutant emissions, which, in the near future, may result in decreased access to potable water, droughts, food shortages, and climate migration on a global scale. Therefore, action aimed at limiting pollutant emissions to the water environment and restoring the quality of polluted water is extremely important. In developed countries, the problem of wastewater emissions to the environment was minimised by transporting waste to treatment plants using elaborate sewage systems or vacuum trucks. As a result of the processes conducted at waste treatment plants, it is possible to remove physical, chemical, and biological pollutants and restore the original quality of water to a state where it is free of substances harmful to living beings. The number and types of stages forming a waste treatment process depend on multiple factors, some of which include the quantity and composition of the waste, the types and concentrations of pollutants in the waste flowing into the treatment plant, and the required level of waste treatment. While municipal wastewater is characterised by a repeatable composition, industrial waste is considerably varied in terms of the type and concentration of pollutants, depending on the type of activity that generates it, the technological advancement of the industrial plant, the application of processes for minimising water consumption, the presence of a local treatment plant providing preliminary waste treatment, and so on. Heavy industry engaged in metal ore processing generates waste characterised by an increased concentration of heavy metals such as, e.g., Cd, Co, Ni, Cu, and V [1]. Electroplating plants, depending on the types of coating produced, generate waste containing, e.g., Ni, Cr, Pb, Cd, and Sn [2,3], as well as cyanides (CN^−^), detergents (anionic, non-ionic, and cationic), etc. On the other hand, waste originating from the tanning industry is characterised by high values of COD and total nitrogen, as well as increased P and Cr contents [4,5,6]. Unlike waste originating from heavy industry, wastewater from the food industry, as well as domestic sewage, is characterised by increased contents of biogenic elements (C, N, P, and S), whose presence in the water environment contributes to eutrophication; therefore, these pollutants need to be removed with high efficiency. In the case of food industry wastewater originating from breweries [7,8,9], dairies [10,11], and sugar factories [12,13], the type of pollutants is similar to those found in domestic sewage, though their concentration is much greater. In the case of treatment plants where the primary pollutant stream includes domestic sewage and food industry wastewater, the use of biological processes, such as denitrification, and chemical processes, like coagulation, is often sufficient to treat the waste. In such situations, the treated waste typically fulfils the requirements defined in legal regulations concerning the maximum concentrations of pollutants for treated waste introduced into municipal sewage systems or into the water and soil.

An element linking the various wastewater treatment technologies is the generation of sewage sludge with a complex composition containing a significant amount of phosphorus compounds, which are an important component of fertilisers used to intensify agricultural production. According to various estimations, the extractive resources of this element will be depleted over the next 50–280 years [14,15], while the location of the richest deposits in just a few countries raises the risk of significant price fluctuations on the market as well as limited availability due to the complex political and economic situation in the future, which has currently made sewage sludge an interesting source of this element that also exhibits a high processing potential, which has made it the subject of numerous studies [16,17]. Sewage sludge is generated, e.g., as a result of coagulation processes that occur after the addition of an appropriate reactant (coagulant) to the treated waste. Commonly applied coagulants include Fe^3+^ and Al^3+^ compounds that undergo hydrolysis after their addition to the wastewater, generating hydrated metal hydroxides with a developed active surface that are capable of adsorbing pollutants or partially binding them as a result of direct chemical reactions.

Ferric chloride (FeCl_3_) was applied in prior research [18], where it was concluded that the most effective (97%) phosphorus removal occurred at a pH of 6.2. The other work [19] applied FeCl_3_ with added tannic acid (C_76_H_52_O_46_) to improve phosphorus removal from wastewater. As a result of the tests conducted at a sewage pH of 7.5, up to 95% of the phosphorus was removed, which was bound in a complex of tannic acid—Fe—P and ferric hydroxyphosphate (Fe_x_(OH)_y_PO_z_). The use of aluminium coagulants such as aluminium chloride (AlCl_3_), aluminium sulphate (Al_2_(SO_4_)_3_∙n H_2_O), or polyaluminium chloride (PAC, Al_n_(OH)_m_Cl_3n−m_) makes it possible to remove up to 74.9% of phosphorus at a pH range of 6.5–8.5, up to 62% at a pH range of 5–9, and even up to 80% at a pH range of 6–8, respectively [20].

Another method for removing phosphorus from wastewater is to precipitate it in the form of poorly soluble salts such as struvite (NH_4_MgPO_4_·6H_2_O), hydroxyapatite (Ca_5_(PO_4_)_3_OH), and amorphous calcium phosphate (Ca_3_(PO_4_)_2_). The precipitation processes occur at high concentrations of NH_4_^+^, Ca^2+^, and PO_4_^3−^ ions and in a pH range of 7.0–10.7. Literature data indicate that the struvite precipitation yield can reach 97%, though it is strongly dependent on the content ratios of P to Mg and P to N. The recovery of phosphorus directly from sewage sludge and eluates is interesting from a scientific and practical perspective, but it generates the risk of introducing pathogens and hazardous organic substances accumulated in the precipitates into the environment. In this case, the sludge must be subjected to hygienisation by applying, e.g., Ca(OH)_2_, CaO, CaO_2_, or 2Na_2_CO_3_·3H_2_O_2_ [21]. A considerable disadvantage of this type of solution is the high cost of the hygienisation processes [22,23].

In the case of more rarely used cerium coagulants that contain Ce^3+^ ions for wastewater treatment (primarily the precipitation of PO_4_^3−^), the pollutant removal process exhibits the highest efficiency at a pH of 7.0–8.5. The removal of PO_4_^3−^ occurs primarily as a result of the direct binding of Ce^3+^ ions with PO_4_^3−^ ions, generating a CePO_4_ precipitate over the course of Reaction (1). Furthermore, the Ce^3+^ cations bind anions such as F^−^, OH^−^, CO_3_^2−^, and C_2_O_4_^2−^ as a result of chemical Reactions (2)–(5) in the form of poorly soluble salts (1)–(5), whose solubility product constants are presented in Table 1.

Ce^3+^ + PO_4_^3−^ → CePO_4_↓ (1)

Ce^3+^ + 3OH^−^ → Ce(OH)_3_↓ (2)

Ce^3+^ + 3F^−^ → CeF_3_↓ (3)

2Ce^3+^ + 3CO_3_^2−^ → Ce_2_(CO_3_)_3_↓ (4)

2Ce^3+^ + 3C_2_O_4_^2−^ → Ce_2_(C_2_O_4_)_3_↓ (5)

When cerium salts are used, the phosphorus concentration in the treated sewage is lower, and the cost of disposal of the resulting sewage sludge is lower (due to the smaller volume of the resulting sludge) than when using iron or aluminium coagulants [32]. The disadvantage of cerium coagulants is their higher purchase cost compared to substances based on iron and aluminium compounds. This fact prompts the search for alternative sources of cerium compounds that could reduce the cost of their purchase and effectively compete with commonly used coagulants.

Research on brewery wastewater treatment using cerium(III) chloride revealed that the process produces sludge with a high content of cerium (101.5 g/kg) and phosphorus (22.2 g/kg), bound primarily in the form of CePO_4_ [9], which suggests the possibility of recovering these elements from the sewage sludge.

This paper presents a processing concept for sewage sludge subjected to extraction using an HCl solution in order to recover cerium and phosphorus and apply them for the preparation of useful products in subsequent stages. The purpose of the tests was to conduct an extraction process and to recover cerium and phosphorus with a high yield, which, in combination with wastewater treatment performed using a cerium(III) chloride solution as a coagulant, could close the cerium circulation within the process. The research presents a new concept of coagulant recovery and complements the gap in research on the recovery of cerium compounds from sewage sludge and fits into the idea of a circular economy.

## 2. Materials and Methods

### 2.1. Materials

**Figure 1:**
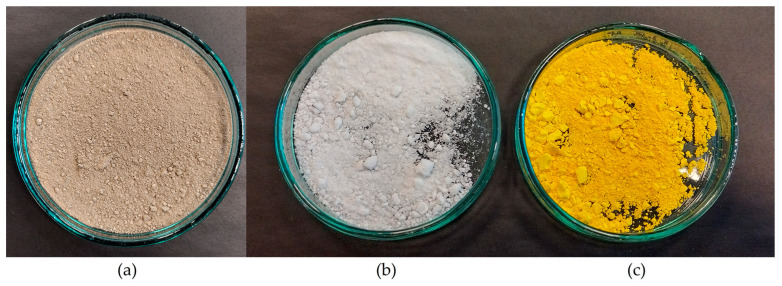
Sewage sludge dried at ambient temperature (**a**) and obtained CeCl_3_·7H_2_O (**b**) and Ce(SO_4_)_2_ (**c**) salts.

The material subjected to testing was sewage sludge obtained by brewery wastewater treatment using a cerium(III) chloride (recovered from spent polishing powder) solution as a coagulant, per the brewery wastewater treatment method described in [9]. Before analysis, the sludge was dried at ambient temperature (20 ± 1 °C) to a solid mass (Figure 1).

The chemical composition of the sewage sludge after mineralisation in aqua regia using a standard laboratory method (HCl:HNO_3_, 3:1, *v*/*v*) is presented in Table 2.

Nitric acid (60%), hydrochloric acid (30%) (Suprapur^®^, Merck, Darmstadt, Germany), sulphuric acid (96%) (analytically pure, Chempur^®^, Piekary Śląskie, Poland), 30% hydrogen peroxide (analytically pure, Chempur^®^, Piekary Śląskie, Poland), sodium hydroxide (analytically pure, Chempur^®^, Piekary Śląskie, Poland), oxalic acid (analytically pure, Warchem Ltd., Zakręt, Poland), a certified multielement standard solution for inductively coupled plasma (ICP) with a concentration of each rare earth element of 50 mg/L (Sigma Aldrich^®^, Saint Louis, MI, USA), and certified multielement standard solutions for ICP with a metal concentration of 10 mg/L and 100 mg/L (AccuStandards^®^, New Haven, CT, USA) were also used in the study. A certified multielement anion standard solution for ion chromatography (IC) (AccuStandards^®^, New Haven, CT, USA) and deionised water with an electrical conductivity of under 0.05 µS/cm (Direct-Q3 UV, Millipore^®^, Burlington, VT, USA) were used as well.

### 2.2. Analytical Methods

The metal and non-metal concentration determination in the water solutions and the mineralised sewage sludge was performed according to standard [33] by inductively coupled plasma optical emission spectrometry (ICP-OES) (Optima 5300DV, Perkin Elmer^®^, Waltham, MA, USA). Inductively coupled plasma mass spectrometry (ICP-MS) (NexION 300S, Perkin Elmer^®^, Waltham, MA, USA) was used to determine cerium content in the water solutions. The measurement was performed according to standard [34]. The metal and non-metal content determination uncertainty was 10%, 15%, 20%, and 25%, depending on the analysed element concentration, with a coverage factor of 2 and a significance level of 95%. The chloride (Cl^−^) determination was performed by ion chromatography according to [35] (DIONEX ICS 5000, Thermo Fisher Scientific^®^, Waltham, MA, USA). The pH measurement was performed per standard [36] using the Inolab pH/ION/Cond 750 multi-parameter meter (WTW^®^, Weilheim, Germany) with a measurement accuracy of ±0.1 pH.

Phase identification was performed by powder X-ray diffraction (DSH) in Bragg-Brentano geometry using a Bruker D8 DISCOVER diffractometer (Bruker^®^, Billerica, MA, USA), CuKα radiation, a Ni filter, and a LYNXEYE XE detector. The mineral composition was determined and calculated based on standards licensed in PDF-4+2022 RDB ICDD (International Centre for Diffraction Data), ICSD (Inorganic Crystal Structure Database), and NIST (National Institute of Standards and Technology). DIFFRAC v.4.2, TOPAS v.4.2, and Bruker AXS software (v.6.0) were applied for registration and diagnostics. The Rietveld methodology was used for the quantitative calculations of crystalline phases and the amorphous substance.

Grain surface morphology and chemical composition in micro-areas were analysed by scanning electron microscopy (SEM) and X-ray energy dispersion spectroscopy (EDS) using an SU3500 SEM microscope (Hitachi^®^, Tokyo, Japan) working in conjunction with an UltraDry EDS Detector (Thermo Fisher Scientific^®^, Waltham, MA, USA) under the following conditions: acceleration voltage—15 keV, detector—BSE, scanning time—40 s, magnification ×1000–×3000. The images were taken after spraying the sample with gold.

### 2.3. Extraction of Cerium and Phosphorus

Central composite design (CCD) and response surface methodology (RSM) were applied to determine the optimal conditions for cerium and phosphorus extraction from the sewage sludge [37,38,39,40]. The optimal input parameters were time (min), the liquid/solid ratio (volume/mass), and the mass of HCl as 100% HCl (mg). The input parameters and the quantities of Ce and P are presented in Table 3.

### 2.4. Precipitation of Solid Cerium(III) Compounds

The extract was obtained under the conditions determined in the optimisation process, i.e., time of 40 min, liquid:solid ratio of 25:1, 330 mg HCl. In order to precipitate Ce_2_(C_2_O_4_)_3_·10H_2_O from the acidic post-extraction solution with a pH of 0.3, oxalic acid (H_2_C_2_O_4_) was added, and then the pH was increased to 1.8 with the addition of NaOH solution [41]. The solution was mixed at 250 rpm for 30 min and set aside for 12 h to obtain a coarse crystalline precipitate [42]. The obtained Ce_2_(C_2_O_4_)_3_·10H_2_O was filtered through a membrane filter (hydrophilic PTFE) with a pore size of 0.45 µm and washed with water to remove the solution residues containing undesired ions. The material was dried at a temperature of 105 ± 1 °C to a solid mass, weighed, and decomposed into CeO_2_ in a muffle furnace at a temperature of 350 ± 5 °C [43]. The obtained CeO_2_ was weighed and subjected to a reaction with HCl (30% m/m) and H_2_O_2_ (30% m/m), mixed and heated to a temperature of about 90 °C [44], after which the solution was filtered, subjected to crystallisation, and dried at 70 ± 1 °C (process no. 1). The second process (process no. 2) involved a reaction of the CeO_2_ with excess H_2_SO_4_ (96% m/m) to obtain Ce_2_(SO_4_)_3_. The reactants were mixed and heated at a temperature of 100 ± 1 °C for an hour, and after heating was concluded, they were diluted with deionised water, and the Ce(IV) was reduced to Ce(III) by the addition of H_2_O_2_ (30%, m/m). The obtained mixture was filtered through a membrane filter (hydrophilic PTFE) with a pore size of 0.45 µm, evaporated, crystallised, and dried at a temperature of 300 ± 5 °C.

The third process (process no. 3) involved a reaction of the CeO_2_ with excess H_2_SO_4_ (96% m/m) to obtain Ce(SO_4_)_2_. The reactants were mixed and heated at a temperature of 100 ± 1 °C for an hour, after which they were heated in a muffle furnace at 300 ± 5 °C, also for an hour, to strip the excess H_2_SO_4_.

### 2.5. Removal of Metals from a Phosphorus-Rich Solution

The removal of metals (Al, Zn, Fe, Cu, and Mn) present in the acidic solution remaining after filtering the Ce_2_(C_2_O_4_)_3_·10H_2_O was conducted by using precipitants such as sodium trithiocarbonate (Na_2_CS_3_), trimercapto-s-triazine, trisodium salt (TMT), and dimethyldithiocarbamate (DMDTC) after the prior increase of the solution pH to 9.5 ± 0.1 [3,45,46]. The obtained solutions were filtered through a PTFE membrane filter with a pore size of 0.45 µm to remove the colloidal sediments and analysed by ICP-OES.

## 3. Results and Discussion

### 3.1. Extraction of Cerium and Phosphate from Sewage Sludge

**Figure 2:**
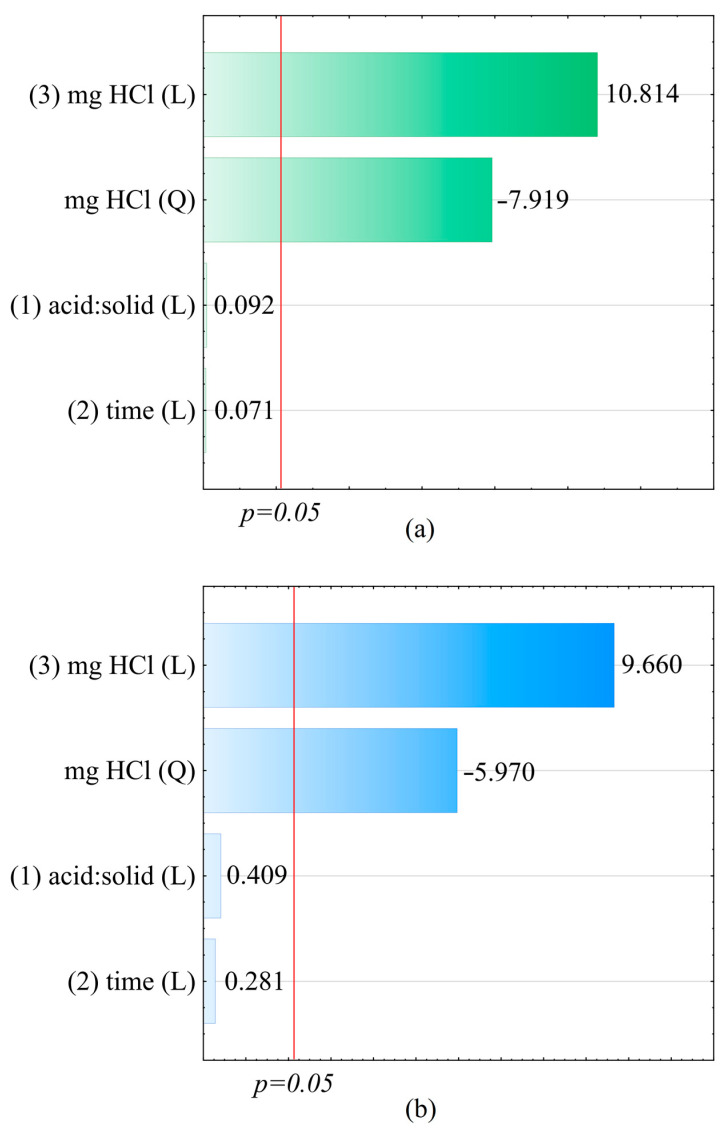
Pareto charts—absolute standardised assessment values for the effects (mg HCl (L), mg HCl (Q), acid:solid (L), and time (L)) for (**a**) Ce^3+^ and (**b**) total P.

The statistical analysis performed (in Tables S1–S4 in Supplementary Materials) showed the lack of significance of some of the independent variables (*p* > 0.05). Further analysis was carried out after excluding insignificant linear–linear interaction, quadratic time, and quadratic liquid:solid ratio effects. Following the optimisation of the Ce and P recovery from sewage sludge containing 10.15% Ce and 2.22% P, it was revealed that the most advantageous process conditions required the application of 350 mg of HCl (as 100% HCl) per 1 g of sludge, an extractant volume of 25 mL, and a reaction time of 40 min. Under the adopted optimal conditions determined by the model, 99.6% of Ce and 97.5% of P were extracted. CCD/RSM was applied in the optimisation process, where the calculated coefficient of determination (R^2^) after reducing the number of statistically insignificant variables was 0.923, and the adjusted R^2^ (R^2^_adj_) was 0.902 (for the cerium extraction optimisation model), which indicated a very good fit of the model to the experimental data. On the other hand, the coefficient of determination (R^2^) calculated for the phosphorus extraction optimisation model was 0.896, while the adjusted R^2^ (R^2^_adj_) was 0.868, which indicated a good fit of the data included in the model to the experimental data. It is typically assumed that if 0 < R^2^ ≤ 0.5, the fit of the model is unsatisfactory; if 0.5 < R^2^ ≤ 0.6, the fit of the model is poor; if 0.6 < R^2^ ≤ 0.8, the fit of the model is satisfactory; if 0.8 < R^2^ ≤ 0.9, the fit of the model is good; and if 0.9 < R^2^ ≤ 1, the fit of the model is very good [47]. Pareto charts (Figure 2) present the standardised effect estimators, which were grouped according to their absolute values. The red line in the chart indicates the significance threshold *p* = 0.05, under which the analysed variables are insignificant. The evaluation of the effects and the results of the ANOVA analysis for Ce^3+^ and P (mg/g) are presented in Table 4, Table 5, Table 6 and Table 7.

**Figure 3:**
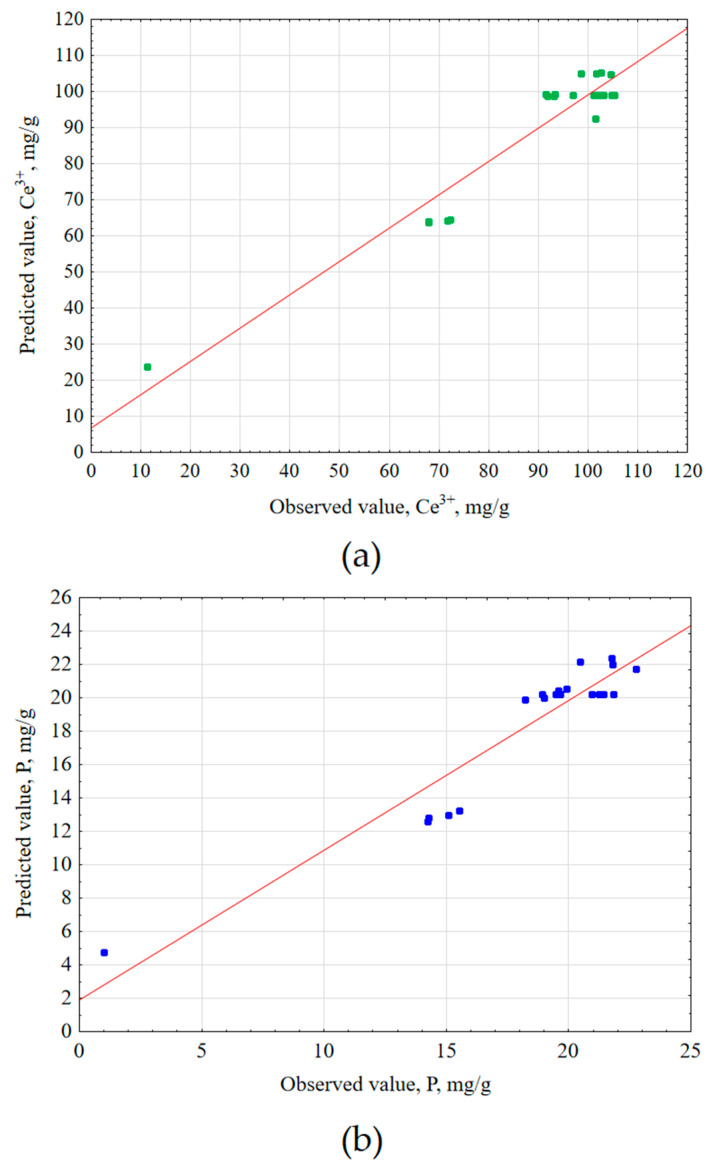
Predicted values vs. observed values for (**a**) Ce^3+^ (R^2^ = 0.923, R^2^_adj_ = 0.902) and (**b**) total P (R^2^ = 0.896, R^2^_adj_ = 0.868) extraction experiments.

Second-order Equations (6) and (7) were determined based on the obtained mathematical models, describing the predicted Ce^3+^ and total P concentrations after extraction. The predicted concentration values were compared to the values obtained experimentally and compiled in Figure 3.

Ce^3+^ conc. = −0.0217 + 0.0069 × (acid:solid) + 0.7113 × (mg HCl) − 0.0012 × (mg HCl)^2^ (6)

total P conc. = −0.2819 + 0.0077 × (acid:solid) + 0.1412 × (mg HCl) − 0.0002 × (mg HCl)^2^ (7)

**Figure 4:**
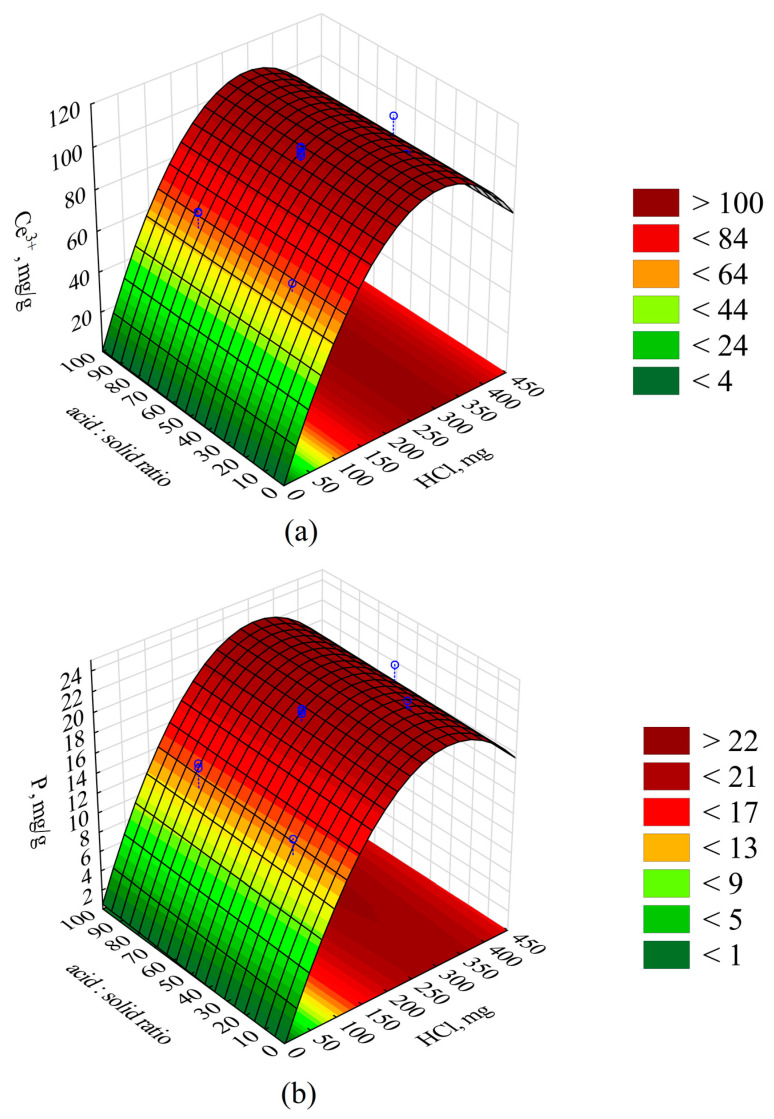
Response surfaces presenting the (**a**) Ce and (**b**) P yield depending on the quantity of the applied HCl and the acid:sludge volume ratio. Blue circles—measured points.

A graphical interpretation of the response surfaces is depicted in the 3D contour plot in Figure 4.

Improving the fit (R^2^ and R^2^_adj_) of both extraction models described would probably be possible by deriving a higher-order equation. This would require taking into account higher-order effects (e.g., cubic effects) [48], which would probably allow for flattening the response surfaces presented in Figure 4 for the mass of HCl used above 300 mg and obtaining the predicted concentrations of Ce and P at a similar level to the measured values. The model, including quadratic effects, causes the surface to curve downward, suggesting a decrease in Ce and P recovery, which deviates from the measured values. As a result of the conducted analysis, it was concluded that the parameter with the greatest influence on the Ce and P extraction efficiency (*p* < 0.05) is the mass of the HCl applied in the process, and a limit value was defined, above which the leaching of cerium and phosphorus is the greatest. From a statistical perspective, the process efficiency was not significantly dependent on the volume ratio of the reaction solution to the mass of the sludge, but the ratio could significantly influence processes at an industrial scale, such as the pumping of the reaction mixture or its transport via pipelines, due to, e.g., the density of the mixture, the resistance of flow, etc. The process duration had no significant influence on the degree of cerium and phosphorus leaching. The high degree of extraction of cerium and phosphorus makes the obtained solution a perfect raw material for the recovery of these elements.

### 3.2. Preparation of Cerium Compounds

An extract with a pH of 0.30 ± 0.01 was obtained following the extraction of cerium (Ce) and phosphorus (P) using an HCl solution under optimal conditions. Table 8 presents the selected physicochemical parameters of the obtained extract.

**Figure 5:**
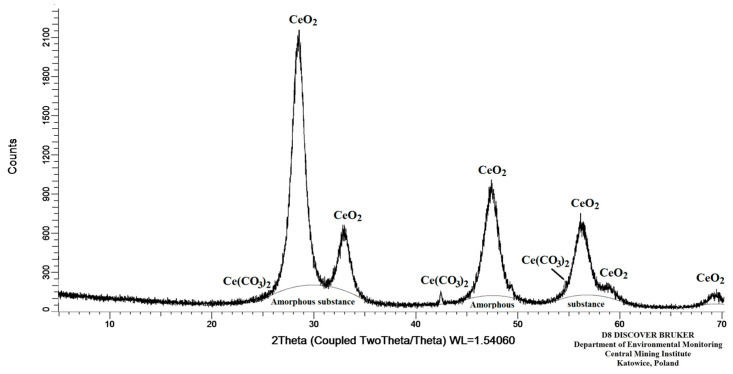
Diffraction pattern of the substance after Ce_2_(C_2_O_4_)_3_·10H_2_O digestion.

The extract contained 3763 mg/L of Ce, which was precipitated after adding an H_2_C_2_O_4_ solution and increasing the pH to 1.8. The Ce concentration determined in the filtrate (1.10 mg/L) revealed that the precipitation process exhibited a yield of 99.97%. A poorly soluble Ce_2_(C_2_O_4_)_3_·10H_2_O precipitate was obtained following the precipitation reaction, which was heated in a muffle furnace at a temperature of 350 ± 5 °C in order to obtain CeO_2_. The low decomposition temperature (350 ± 5 °C) was selected to ensure that the obtained CeO_2_ would be characterised by a small particle size and thereby a greater reactivity towards acids. The crystalline composition of the obtained compound was analysed by XRD as described in the Analytical Methods section and is presented in Table 9, whereas a diffractogram of this compound is depicted in Figure 5. 

At the same time, the test results revealed explicitly that the primary component of the obtained material was CeO_2_ (74.0%), while 24.5% was an amorphous phase, which could also be CeO_2_ [43,49,50]. The performed calculations demonstrated that the mass of the obtained oxide constituted 99.5% of the theoretical mass. 

Three independent processes were conducted to obtain cerium salts. The first process yielded CeCl_3_·7H_2_O by mixing CeO_2_ with excess HCl (30%, m/m) and H_2_O_2_ (30%, m/m) [9,44], and heating the solution to a temperature of about 90 ± 1 °C, followed by evaporation and crystallisation (Figure 1). Under these conditions, CeCl_3_·7H_2_O was obtained with a yield of 97.0%. A sample of the obtained substance was subjected to chemical analysis, which revealed that Ce constituted 98.6% of the analysed cations (30 elements).

The second process yielded Ce_2_(SO_4_)_3_ by adding excess concentrated H_2_SO_4_ (96%, m/m) to the CeO_2_, mixing and heating at a temperature of 100 ± 1 °C for 60 ± 1 min, followed by diluting with deionised water and reducing the Ce(IV) to Ce(III) by adding H_2_O_2_ (30%, m/m) (Figure 1). The obtained mixture was filtered through a membrane filter (hydrophilic PTFE) with a pore size of 0.45 µm, evaporated, crystallised, and dried at a temperature of 300 ± 5 °C. In this case, Ce_2_(SO_4_)_3_ was obtained with a yield of 97.4%, while a chemical analysis confirmed that Ce constituted 95.9% of all the analysed cations (30 elements) in the Ce_2_(SO_4_)_3_ sample.

The third process yielded Ce(SO_4_)_2_ by subjecting the obtained CeO_2_ to a reaction with excess concentrated H_2_SO_4_ (96%, m/m) relative to the required stoichiometric quantity and heating at a temperature of 100 ± 1 °C, followed by heating in a muffle furnace at a temperature of 300 ± 5 °C. The process exhibited a Ce(SO_4_)_2_ yield of 98.3%. The conducted chemical analysis of the obtained compound confirmed that Ce constituted 97.5% of all the analysed cations (30 elements).

**Figure 6:**
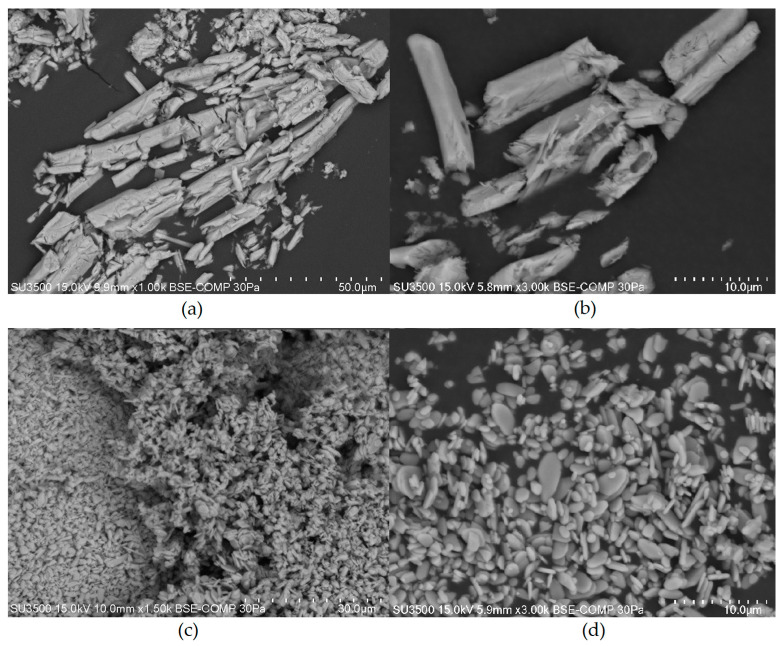
SEM images obtained for Ce_2_(SO_4_)_3_ at a magnification of ×1000 (**a**) and a magnification of ×3000 (**b**), and for Ce(SO_4_)_2_ at a magnification of ×1500 (**c**) and a magnification of ×3000 (**d**).

An analysis of the obtained CeCl_3_·7H_2_O, Ce_2_(SO_4_)_3_ and Ce(SO_4_)_2_ compositions performed by SEM-EDS, ICP-OES, and IC is presented in Table 10. SEM images obtained for Ce_2_(SO_4_)_3_ and Ce(SO_4_)_2_ are presented in Figure 6. The analysis results confirm the expected obtained cerium salt composition, as the percentage elemental content calculated based on the results of the conducted tests for individual salts exhibits minor differences from the theoretical values calculated based on the chemical formulas of these compounds. All stages of the recovery of each cerium salt were carried out with high efficiency, which resulted in high efficiency throughout the entire process. Furthermore, it can be assumed that expanding the process from the laboratory to a semi-commercial or commercial scale will make it possible to improve the process yield by preventing material losses associated with the conduct of experiments at a small laboratory scale. Analysing the quantity of pollutants in the obtained salts makes it possible to classify them as technically pure substances; however, if they were to be applied as active substances of cerium coagulants, the purity of the obtained compounds could be deemed sufficient.

Cerium salts obtained in a complex and environmentally harmful production process are an alternative to iron and aluminium compounds but are 3–6 times more expensive [51,52]. Therefore, recovering them from industrial waste such as spent polishing powders (16,000 tons per year; [53]), spent catalysts [54,55] can contribute to a significant reduction in their prices. According to the European Commission reports from 2017–2023, Light Rare Earth Elements (LREE) recycling remains at an extremely low level of approximately 1% [56,57,58]. Reducing the costs of cerium compounds at the stage of installation operation can also be achieved through the high-efficiency recycling of sewage sludge described in this article, thus largely closing the cerium cycle in the process. Cerium salts needed for the start-up of the installation and to cover any process losses can be replenished with recycled salts [9,44,59]. 

### 3.3. Removal of Metals from a Phosphorus-Rich Solution and the Possible Directions of Its Processing and Application

Phosphatic fertilisers for application in agriculture must be characterised by an appropriate level of purity, and they must particularly be free of heavy metals, whose elevated concentrations could negatively impact the environment. In the case of the studied phosphorus-rich eluate, it was revealed to contain metals (Al, Cu, Zn, Fe, and Mn), whose concentrations had to be reduced to safe values. Additional experiments were performed based on literature data [2,3] describing the high efficiency of heavy metal removal from acidic eluates through the application of DMDTC, TMT, and Na_2_CS_3_ solutions. The metal removal was conducted using four methods, i.e., ncreasing the pH to 9.5; increasing the pH to 9.5 and applying stoichiometric quantities of one of the three solutions relative to the metal contents (Al, Cu, Zn, Fe, and Mn), i.e., TMT, DMDTC, and Na_2_CS_3_. Table 11 presents the results of these tests.

The application of DMDTC yielded an eluate characterised by the lowest concentration of metal cations. Therefore, the initial alkalising of the eluate (pH 9.5) and the application of a stoichiometric dose of DMDTC relative to the concentrations of the individual metal cations should be considered the most effective method of their removal. The phosphate solution obtained after applying NaOH and DMDTC was characterised by a low metal concentration, which is why it may constitute a valuable resource for obtaining phosphate salts by the application of, e.g., a CaCl_2_ solution in an alkaline medium (pH correction is not required due to the alkaline reaction of the post-process solution obtained after removing the metal cations in the form of poorly soluble colloidal precipitates), retaining a 1.5 molar ratio of Ca:P. Furthermore, rinsing the calcium phosphate precipitate with an NaOH solution may remove pollutants such as Al [60]. Another method of obtaining useful phosphate salts may consist of precipitating struvite (MgNH_4_PO_4_·6H_2_O). As demonstrated by prior research [61], increasing the Mg:P ratio may have a beneficial influence on the precipitation of these phosphorus compounds, while conducting the precipitation process at a pH above 9.0 reduces the negative influence of the humic acids that may potentially be present in the solution.

Another work [62] presented the positive influence of brewery sewage sludge applied to increase the agricultural production of maize as a result of supplying the soil with elements such as nitrogen (N), phosphorus (P), and sulphur (S). A problem with the application of this material was the increase in the concentration of lead (Pb) in the soil, which over time may lead to the accumulation of this element and result in exceeded permissible levels of lead (Pb) in the soil and its accumulation in cultivated plant tissue, and consequently in obtaining agricultural products potentially hazardous to health. In this work, as a result of increased pH and the application of DMDTC, the concentrations of Fe, Mn, Al, Cu, and Zn were lowered by 93.8%, 95.4%, 88.6%, 96.8%, and 90.0%, respectively. Therefore, the potential heavy metal contamination risk for the synthesised phosphorus salts (obtained per one of the methods described above) is negligible. Additionally, the performed tests demonstrated that the lead concentration in the filtrate after filtering the Ce_2_(C_2_O_4_)_3_·10H_2_O as well as after applying the precipitants (NaOH and DMDTC) was <0.005 mg/L and was therefore negligible as a potential pollutant in the phosphate salts obtained using the solution remaining after the prior separation of Ce_2_(C_2_O_4_)_3_·10H_2_O. Nevertheless, the presence of undesired metals in the sewage sludge and the solutions after their processing is possible, and in the event of their occurrence, action should be taken to reduce their concentrations to safe levels.

### 3.4. Potential Application of the Precipitates and Post-Reaction Solutions

The precipitate, composed primarily of organic matter unaffected by the process described in pt. 3.1, could probably be applied for fertilisation purposes, e.g., for degraded post-industrial land reclamation, though it is necessary to perform a series of additional tests regarding its potential biological and chemical risks, which would explicitly determine the usefulness of this material for the aforementioned applications, but this is outside the scope of this work. However, this study can certainly inspire further interesting research. Prior work [21] demonstrated the usefulness of materials of biological origin in the process of post-industrial land reclamation, but only after the sludge was subjected to hygienisation, which reduced the quantity of hazardous bacteria by means of calcium peroxide (CaO_2_) or sodium percarbonate (2Na_2_CO_3_·3H_2_O_2_), compared to the commonly applied calcium hydroxide (Ca(OH)_2_), calcium oxide (CaO), and calcium carbonate (CaCO_3_). Such a process may also be necessary in the case of the precipitate obtained after the extraction of sewage sludge using an HCl solution. Furthermore, if necessary, the precipitate composition may be modified to increase the concentration of phosphorus originating from the phosphate salts that could be obtained in the processes described in Section 3.3.

The final solution obtained after all the processing stages contained a high quantity of chloride ions and sodium (about 36 g/L NaCl). However, crystalline sodium chloride (NaCl) can be separated from this post-process solution by applying a water desalination process, such as multi-stage flash distillation (MSF) [63], reverse osmosis (RO) preceded by other concentration processes [64], or water evaporation in evaporation ponds [65], and using the crystallised salt as an additive to the road salt used for de-icing. As in the case of extraction sediments, the usefulness of salt should be determined by conducting tests for the content of chemical and biological pollutants.

## 4. Conclusions

Following an analysis of the chemical composition of brewery sewage sludge obtained by coagulation using recycled CeCl_3_, it was demonstrated that due to the high concentration of Ce and P (101.5 g/kg Ce and 22.2 g/kg P), the waste may serve as a potential raw material for the recovery of these elements and may be subjected to recovery processes. The application of CCD/RSM enabled the determination of optimal extraction process conditions, i.e., 0.35 g HCl as 100% HCl per 1 g of sludge, 40 min reaction time, and an extractant volume of 25 mL per 1 g, with extraction efficiency of 99.6% and 97.5% for Ce and P, respectively. The tests demonstrated excellent recovery of cerium (97.0–98.3%) in the form of cerium chlorides or sulphates, as well as a very good extraction of phosphorus (97.5%), which may be subjected to further processing by precipitating struvite, hydroxyapatite, and calcium phosphate. Based on the performed tests, it can be concluded that the discussed processing concept exhibits potential for development, and the processes could be enhanced for conduction at a greater scale. The by-products obtained in the process, such as a precipitate containing organic matter and a post-process solution with elevated salinity, can find industrial application, but only after the conduction of additional tests and experiments necessary from the perspective of their potential use, which certainly constitutes a field of new, interesting, and comprehensive research related to the recovery of phosphorus from sewage sludge in the context of the prior utilisation of modern coagulants, where the active substances may include cerium, zirconium, or titanium compounds. The proposed method of sewage sludge processing is a new and innovative way of repurposing this waste, where unit processes yield a number of products that could potentially be reused in, e.g., municipal or industrial waste treatment, degraded industrial land reclamation, traffic (road de-icing), and even agriculture. Furthermore, it should be stressed that the presented recovery process concept, together with the proposed applications of individual products and by-products, offers an excellent contribution to the idea of a circular economy.

## Figures and Tables

**Table 1:** Solubility product constants (K_SP_) of selected cerium compounds.

Compound	Solubility Product Constant, K_SP_	Source
CePO_4_	1.00 × 10^−23^	[24]
	10^−24.3^	[25]
	10^−26.27^	[26]
Ce(OH)_3_	1.6 × 10^−20^	[24]
	10^−21^	[27]
CeF_3_	8.00 × 10^−16^	[24]
	1.4 × 10^−18^	[28]
Ce_2_(CO_3_)_3_	10^−21.80^	[29]
Ce_2_(C_2_O_4_)_3_	1.84 × 10^−28^	[30]
	5.9 × 10^−30^	[31]

**Table 2:** Selected physicochemical parameters of the sludge used in the study.

Parameter	Unit	Result ± Measurement Uncertainty
Cerium (Ce)	g/kg	101.5 ± 10
Total Phosphorus (P)	g/kg	22.2 ± 2.2
Sodium (Na)	g/kg	3.65 ± 0.37
Magnesium (Mg)	g/kg	1.05 ± 0.11
Calcium (Ca)	g/kg	21.3 ± 2.1
Iron (Fe)	g/kg	3.46 ± 0.35
Manganese (Mn)	g/kg	0.10 ± 0.02
Aluminium (Al)	g/kg	1.23 ± 0.12
Barium (Ba)	g/kg	0.185 ± 0.037
Strontium (Sr)	g/kg	0.084 ± 0.021
Zinc (Zn)	g/kg	0.417 ± 0.083

**Table 3:** Empirical conditions for CCD/RSM and the results (Ce and P) for sewage sludge (HCl 35.0–405.0 mg, liquid:solid mass ratio (7.96–92.0:1), time 6.36–73.6 min); *—centre of the plan.

	Experimental Conditions			Experimental Results	
Run	Liquid:Solid Ratio, x_1_	Time (min), x_2_	HCl, mg, x_3_	P, mg/g	Ce^3+^, mg/g
1	25.0	20.0	110	14.24 ± 2.14	67.95 ± 10.19
2	25.0	20.0	330	22.73 ± 3.41	104.5 ± 15.68
3	25.0	60.0	110	14.27 ± 2.14	67.86 ± 10.18
4	25.0	60.0	330	21.80 ± 3.27	101.6 ± 15.24
5	75.0	20.0	110	15.09 ± 2.26	71.73 ± 10.76
6	75.0	20.0	330	20.48 ± 3.07	98.54 ± 14.78
7	75.0	60.0	110	15.54 ± 2.33	72.23 ± 10.84
8	75.0	60.0	330	21.76 ± 3.26	102.6 ± 15.39
9	7.96	40.0	220	18.23 ± 2.73	91.89 ± 13.78
10	92.0	40.0	220	19.91 ± 2.99	91.44 ± 13.72
11	50.0	6.36	220	18.99 ± 2.85	93.18 ± 13.98
12	50.0	73.6	220	19.58 ± 2.94	93.35 ± 14.00
13	50.0	40.0	35	1.014 ± 0.152	11.17 ± 1.68
14	50.0	40.0	405	21.84 ± 3.28	101.4 ± 15.22
15 *	50.0	40.0	220	19.67 ± 2.95	97.03 ± 14.55
16 *	50.0	40.0	220	20.96 ± 3.14	101.1 ± 15.16
17 *	50.0	40.0	220	21.23 ± 3.18	103.2 ± 15.47
18 *	50.0	40.0	220	21.42 ± 3.21	105.3 ± 15.80
19 *	50.0	40.0	220	19.45 ± 2.92	104.8 ± 15.72
20 *	50.0	40.0	220	18.93 ± 2.84	102.2 ± 15.33

**Table 4:** Analysis of the central composite design experiment using Statistica 13. Evaluation of the effects, Ce^3+^; R^2^ = 0.923; R^2^_adj_ = 0.902, MS = 48.844.

Parameter	Effect	Standard Error	t(15)	*p*	CI (−95%)	CI (+95%)	Factor	Standard Error	CI (−95%)	CI (+95%)
Constant value	99.022	1.999	49.547	<0.001	94.762	103.281	99.022	1.999	94.762	103.281
(1) liquid:solid (L) *	0.347	3.782	0.092	0.928	−7.715	8.408	0.173	1.891	−3.858	4.204
(2) time (L)	0.270	3.782	0.071	0.944	−7.792	8.332	0.135	1.891	−3.896	4.166
(3) mg HCl (L)	40.904	3.782	10.814	<0.001	32.842	48.966	20.452	1.891	16.421	24.483
mg HCl (Q)	−28.896	3.649	−7.919	<0.001	−36.674	−21.119	−14.448	1.824	−18.337	−10.560

*—mass ratio, statistically significant if *p* < 0.05.

**Table 5:** ANOVA analysis results for Ce^3+^ extraction.

Parameter	SS	df	MS	F	*p*
(1) liquid:solid (L) *	0.410	1	0.410	0.008	0.928
(2) time (L)	0.248	1	0.248	0.005	0.944
(3) mg HCl (L)	5712.425	1	5712.425	116.952	<0.001
mg HCl (Q)	3063.310	1	3063.310	62.716	<0.001
Error	732.661	15	48.844		
Total sum of square	9509.054	19			

*—mass ratio, ANOVA model coefficients after excluding insignificant linear–linear interaction, quadratic time, and quadratic liquid:solid ratio effects, statistically significant if *p* < 0.05.

**Table 6:** Analysis of the central composite design experiment using Statistica 13. Evaluation of the effects, total P; R^2^ = 0.896; R^2^_adj_ = 0.868, MS = 3.081.

Parameter	Effect	Standard Error	t(15)	*p*	CI (−95%)	CI (+95%)	Factor	Standard Error	CI (−95%)	CI (+95%)
Constant value	20.226	0.502	40.295	<0.001	19.156	21.296	20.226	0.502	19.156	21.296
(1) liquid:solid (L) *	0.389	0.950	0.409	0.688	−1.636	2.413	0.194	0.475	−0.818	1.207
(2) time (L)	0.267	0.950	0.281	0.783	−1.758	2.291	0.133	0.475	−0.879	1.146
(3) mg HCl (L)	9.177	0.950	9.660	<0.001	7.152	11.202	4.589	0.475	3.576	5.601
mg HCl (Q)	−5.471	0.916	−5.970	<0.001	−7.424	−3.518	−2.736	0.458	−3.712	−1.759

*—mass ratio, statistically significant if *p* < 0.05.

**Table 7:** ANOVA analysis results for total P extraction.

Parameter	SS	df	MS	F	*p*
(1) liquid:solid (L) *	0.515	1	0.515	0.167	0.688
(2) time (L)	0.243	1	0.243	0.079	0.783
(3) mg HCl (L)	287.540	1	287.540	93.325	<0.001
mg HCl (Q)	109.810	1	109.810	35.640	<0.001
Error	46.216	15	3.081		
Total sum of square	444.324	19			

*—mass ratio, ANOVA model coefficients after excluding insignificant linear–linear interaction, quadratic time, and quadratic liquid:solid ratio effects, statistically significant if *p* < 0.05.

**Table 8:** Selected chemical parameters of the extract obtained after acid leaching.

Parameter	Unit	Result ± Measurement Uncertainty
pH	-	0.30 ± 0.01
Cerium (Ce)	mg/L	3763 ± 376
Total Phosphorus (P)	mg/L	790 ± 79
Sodium (Na)	mg/L	149 ± 15
Magnesium (Mg)	mg/L	36.4 ± 3.6
Calcium (Ca)	mg/L	774 ± 77
Iron (Fe)	mg/L	96.7 ± 9.7
Aluminium (Al)	mg/L	43.6 ± 4.4
Zinc (Zn)	mg/L	14.3 ± 1.4

**Table 9:** Chemical composition of the substance after Ce_2_(C_2_O_4_)_3_·10H_2_O digestion (XRD analysis).

Parameter	Unit	Result ± Measurement Uncertainty
CeO_2_	%	74.0 ± 1.0
Ce(CO_3_)_2_	%	1.0 ± 1.0
Amorphous substance	%	24.5 ± 0.5

**Table 10:** Chemical composition of the obtained cerium salts.

Salt	Parameter	Unit	Result ± Measurement Uncertainty		
			Theoretical Content	ICP—OES, IC *	SEM—EDS
CeCl_3_·7H_2_O	Ce	%	37.62	37.21 ± 3.72	-
	Cl	%	28.55	29.15 ± 2.92 *	-
Ce_2_(SO_4_)_3_	Ce	%	49.30	46.61 ± 4.66	49.6
	S	%	16.92	-	16.1
	O	%	33.78	-	33.2
	SO_4_^2−^	%	50.70	52.52 ± 5.25	-
Ce(SO_4_)_2_	Ce	%	42.17	41.32 ± 4.13	44.0
	S	%	19.30	-	19.3
	O	%	38.52	-	36.5
	SO_4_^2−^	%	57.82	58.68 ± 5.87	-

* determination made using ion chromatography technique.

**Table 11:** Selected chemical parameters of the extract following metal removal by various methods.

Parameter	Unit	Result ± Measurement Uncertainty				
		The Solution after Filtration Ce_2_(C_2_O_4_)_3_·10H_2_O	NaOH	TMT	DMDTC	Na_2_CS_3_
pH	-	1.8 ± 0.1	9.5 ± 0.1	9.5 ± 0.1	9.5 ± 0.1	9.5 ± 0.1
Total Phosphorus (P)	mg/L	561 ± 56	453 ± 45	442 ± 44	438 ± 44	440 ± 44
Copper (Cu)	mg/L	2.20 ± 0.22	1.82 ± 0.18	0.829 ± 0.166	0.071 ± 0.018	1.97 ± 0.20
Iron (Fe)	mg/L	61.0 ± 6.1	44.68 ± 4.47	3.17 ± 0.32	1.85 ± 0.19	1.15 ± 0.12
Manganese (Mn)	mg/L	2.33 ± 0.23	0.22 ± 0.04	0.241 ± 0.048	0.106 ± 0.021	0.136 ± 0.027
Aluminium (Al)	mg/L	49.7 ± 5.0	1.66 ± 0.17	8.40 ± 0.84	5.66 ± 0.57	2.67 ± 0.27
Zinc (Zn)	mg/L	9.71 ± 0.97	0.76 ± 0.15	1.34 ± 0.13	0.97 ± 0.19	1.12 ± 0.11
Lead (Pb)	mg/L	<0.005 ± 0.001	<0.005 ± 0.001	<0.005 ± 0.001	<0.005 ± 0.001	<0.005 ± 0.001

## Data Availability

Data are contained within the article and Supplementary Materials.

## Supplementary Materials

The following supporting information can be downloaded at: https://www.mdpi.com/article/10.3390/ma17040938/s1, Table S1. Analysis of the central composite design experiment using Statistica 13. Evaluation of the effects, Ce^3+^; R^2^ = 0.938; R^2^_adj_ = 0.882, MS = 59.277. Table S2. ANOVA analysis results for Ce^3+^ extraction. Table S3. Analysis of the central composite design experiment using Statistica 13. Evaluation of the effects, total P; R^2^ = 0.904; R^2^_adj_ = 0.817, MS = 4.287. Table S4. ANOVA analysis results for total P extraction.

## Abbreviations

The following abbreviations are used in this manuscript:

CCD	Central composite design
COD	Chemical oxygen demand
IC	Ion chromatography
ICP-MS	Inductively coupled plasma mass spectrometry
ICP-OES	Inductively coupled plasma optical emission spectrometry
RSM	Response surface methodology
Total P	Total phosphorus as P
