# Supplementary material for: Recovery of Cerium Salts from Sewage Sludge Resulting from the Coagulation of Brewery Wastewater with Recycled Cerium Coagulant
Source: Materials (Basel). 2024 Feb 17;17(4):938. doi: 10.3390/ma17040938 (PMC10889923; doi:10.3390/ma17040938)
Supplement: Supplementary file 1 [file materials-17-00938-s001.zip › materials-2838646-supplementary.pdf]

materials MDPI

Supplementary Materials

Recovery of Cerium Salts from Sewage Sludge Resulting from the Coagulation of Brewery Wastewater with Recycled Cerium Coagulant

Paweł Lejwoda [1], Barbara Białecka [2], Krzysztof Barbusiński [3] and Maciej Thomas [4,*]

[1] Department of Energy Saving and Air Protection, Central Mining Institute in Katowice, Plac Gwarków 1, 40-166 Katowice, Poland; plejwoda@gig.eu
[2] Department of Environmental Monitoring, Central Mining Institute in Katowice, Plac Gwarków 1, 40-166 Katowice, Poland; bbialecka@gig.eu
[3] Department of Water and Wastewater Engineering, Silesian University of Technology, Konarskiego 18, 44-100 Gliwice, Poland; krzysztof.barbusinski@polsl.pl
[4] Faculty of Environmental Engineering and Energy, Cracow University of Technology, Warszawska 24, 31-155 Cracow, Poland
* Correspondence: maciej.thomas@pk.edu.pl

Table S1. Analysis of the central composite design experiment using Statistica 13. Evaluation of the effects, Ce^{3+}; $R^2 = 0.938$; $R^2_{adj} = 0.882$, MS = 59.277.

Parameter	Effect	Standard error	t(10)	p	CI (-95%)	CI (+95%)	Factor	Standard error	CI (-95%)	CI (+95%)
Constant value	102.050	3.140	32.499	< 0.001	95.053	109.046	102.050	3.140	95.053	109.046
(1) liquid:solid (L)	0.347	4.167	0.083	0.935	-8.937	9.631	0.173	2.083	-4.469	4.815
liquid:solid (Q)	-4.633	4.056	-1.142	0.280	-13.670	4.405	-2.316	2.028	-6.835	2.203
(2) time (L)	0.270	4.167	0.065	0.950	-9.014	9.554	0.135	2.083	-4.507	4.777
time (Q)	-3.501	4.056	-0.863	0.408	-12.539	5.536	-1.751	2.028	-6.270	2.768
(3) mg HCl (L)	40.904	4.167	9.817	< 0.001	31.620	50.188	20.452	2.083	15.810	25.094
mg HCl (Q)	-29.631	4.056	-7.305	< 0.001	-38.669	-20.593	-14.816	2.028	-19.334	-10.297
1L relative to 2L	1.887	5.444	0.347	0.736	-10.243	14.017	0.944	2.722	-5.122	7.009
1L relative to 3L	-3.296	5.444	-0.605	0.558	-15.426	8.834	-1.648	2.722	-7.713	4.417
2L relative to 3L	0.178	5.444	0.033	0.975	-11.952	12.308	0.089	2.722	-5.976	6.154

* mass ratio, statistically significant if $p < 0.05$.

Table S2. ANOVA analysis results for Ce^{3+} extraction.

Parameter	SS	df	MS	F	p
(1) liquid:solid (L)*	0.410	1	0.410	0.007	0.935
liquid:solid (Q)*	77.323	1	77.323	1.304	0.280
(2) time (L)	0.248	1	0.248	0.004	0.950
time (Q)	44.167	1	44.167	0.745	0.408
(3) mg HCl (L)	5712.425	1	5712.425	96.369	< 0.001
mg HCl (Q)	3163.284	1	3163.284	53.365	< 0.001
1L relative to 2L	7.123	1	7.123	0.120	0.736
1L relative to 3L	21.731	1	21.731	0.367	0.558
2L relative to 3L	0.063	1	0.063	0.001	0.975
Error	592.765	10	59.277		
Total sum of square	9509.054	19			

* mass ratio, statistically significant if $p < 0.05$.

Table S3. Analysis of the central composite design experiment using Statistica 13. Evaluation of the effects, total P; $R^2 = 0.904$; $R^2_{adj} = 0.817$, MS = 4.287.

Parameter	Effect	Standard error	t(10)	p	CI (-95%)	CI (+95%)	Factor	Standard error	CI (-95%)	CI (+95%)
Constant value	20.217	0.844	23.942	< 0.001	18.336	22.099	20.217	0.844	18.336	22.099
(1) liquid:solid (L)	0.389	1.120	0.347	0.736	-2.108	2.885	0.194	0.560	-1.054	1.443
liquid:solid (Q)	-0.065	1.091	-0.059	0.954	-2.495	2.366	-0.032	0.545	-1.247	1.183
(2) time (L)	0.267	1.120	0.238	0.817	-2.230	2.763	0.133	0.560	-1.115	1.382
time (Q)	0.088	1.091	0.081	0.937	-2.343	2.518	0.044	0.545	-1.171	1.259
(3) mg HCl (L)	9.177	1.120	8.190	< 0.001	6.680	11.674	4.589	0.560	3.340	5.837
mg HCl (Q)	-5.469	1.091	-5.014	< 0.001	-7.899	-3.039	-2.734	0.545	-3.950	-1.519
1L relative to 2L	0.655	1.464	0.447	0.664	-2.607	3.917	0.327	0.732	-1.304	1.958
1L relative to 3L	-1.105	1.464	-0.755	0.468	-4.367	2.157	-0.553	0.732	-2.184	1.078
2L relative to 3L	-0.034	1.464	-0.023	0.982	-3.296	3.228	-0.017	0.732	-1.648	1.614

* mass ratio, statistically significant if $p < 0.05$.

Table S4. ANOVA analysis results for total P extraction.

Parameter	SS	df	MS	F	p
(1) liquid:solid (L)*	0.515	1	0.515	0.120	0.736
liquid:solid (Q)*	0.015	1	0.015	0.003	0.954
(2) time (L)	0.243	1	0.243	0.057	0.817
time (Q)	0.028	1	0.028	0.006	0.937
(3) mg HCl (L)	287.540	1	287.540	67.080	< 0.001
mg HCl (Q)	107.758	1	107.758	25.139	< 0.001
1L relative to 2L	0.858	1	0.858	0.200	0.664
1L relative to 3L	2.443	1	2.443	0.570	0.468
2L relative to 3L	0.002	1	0.002	0.001	0.982
Error	42.865	10	4.287		
Total sum of square	444.324	19			

* mass ratio, statistically significant if $p < 0.05$.
